# Magnesium Replacement Does Not Improve Insulin Resistance in Patients With Metabolic Syndrome: A 12-Week Randomized Double-Blind Study

**DOI:** 10.14740/jocmr1580w

**Published:** 2014-09-09

**Authors:** Maria de Lourdes Lima de Souza e Silva, Thomaz Cruz, Luiz Erlon Rodrigues, Ana Marice Ladeia, Olivia Bomfim, Lucas Olivieri, Juliana Melo, Raquel Correia, Mirna Porto, Alexandre Cedro

**Affiliations:** a Escola Bahiana de Medicina e Saude Publica, FBDC, Salvador, Bahia, Brasil; b Universidade Salvador, UNIFACS, Salvador, Bahia, Brasil; c Departmento de Medicina, Programa de Pos-graduacao em Medicina e Saude da Faculdade de Medicina da Bahia, Universidade Federal da Bahia, Brasil

**Keywords:** Metabolic syndrome, Magnesium replacement, Magnesium and insulin resistance

## Abstract

**Background:**

To evaluate the effect of magnesium (Mg) replacement on insulin resistance and cardiovascular risk factors in women with metabolic syndrome (MS) without diabetes.

**Methods:**

This 12-week clinical randomized double-blind study compared the effects of 400 mg/day of Mg with those of a placebo (n = 72) on fasting glucose, insulin, HOMA-IR, lipid profile and CRP. Mg was measured in serum (SMg) and in mononuclear cells (MMg).

**Results:**

Hypomagnesemia (SMg < 1.7 mg/dL) was seen in 23.2% of patients and intracellular depletion in 36.1% of patients. The MMg means were lower in patients with obesity (0.94 ± 0.54 μg/mg vs. 1.19 ± 0.6 μg/mg, P = 0.04), and insulin resistance (0.84 ± 0.33 μg/mg vs. 1.14 ± 0.69 µg/mg, P < 0.05). Mg replacement did not alter SMg (1.82 ± 0.14 mg/dL vs. 1.81 ± 0.16 mg/dL, P = 0.877) and tended to increment MMg (0.90 ± 0.40 μg/mg vs. 1.21 ± 0.73 μg/mg, P = 0.089). HOMA-IR did not alter in interventions nor in placebo group (3.2 ± 2.0 to 2.8 ± 1.9, P = 0.368; 3.6 ± 1.9 to 3.2 ± 1.8, respectively), neither did other metabolic parameters.

**Conclusion:**

Serum and intracellular Mg depletion is common in patients with MS; however, Mg replacement in recommended dosage did not increase significantly Mg levels, neither reduced insulin resistance or metabolic control.

## Introduction

An association between obesity, high fasting triglycerides, impaired glucose tolerance, hypertension and cardiovascular disease, has been recognized for a long time. These major risk factors tend to cluster together in many individuals, having insulin resistance as a main pathophysiological linkage [1], which was called metabolic syndrome (MS), a complex entity related to metabolic and cardiovascular risk factors and pro-thrombotic and pro-inflammatory states.

The whole comprehension of the insulin resistance pathogenesis is still in question. Magnesium (Mg), a predominant intracellular ion, is a crucial metallic co-factor of many enzymatic reactions involved in post-receptor signaling and is critically involved in energy metabolism, fatty acid synthesis and glucose utilization [2]. Some authors suggest that Mg deficiency can contribute to insulin resistance [3], and epidemiological studies have described an inverse relationship between the intake levels of Mg rich foods and MS [4].

Hypomagnesemia has been seen in obese children [5, 6] and in people with MS [7]. Other studies demonstrate some benefits of Mg replacement in people with diabetes [8, 9], and those with insulin resistance [10].

The objective of this study was to evaluate the effect of Mg replacement on insulin resistance and on other cardiovascular risk factors in patients with MS without diabetes mellitus.

## Material and Methods

A 12-week clinical randomized double-blind placebo-controlled trial, with 72 outpatients in an obesity reference center, was conducted.

The inclusion criteria for patients were they had to be between the ages of 21 and 70 years, with MS as defined by the International Diabetes Federation criteria that require the presence of abdominal obesity, defined by waist circumference ≥ 90 cm in men and ≥ 80 cm in women, for South American subjects, and two additional criteria, as follows: 1) hypertriglyceridemia ≥ 150 mg/dL; 2) HDL cholesterol < 50 mg/dL in women; 3) high blood pressure: ≥ 130/≥ 85 mm Hg; 4) fasting glucose ≥ 100 mg/dL. Individuals on fibrates, on anti-hypertensive or anti-diabetic therapy were included in criteria 1, 3 and 4 respectively [11]. Patients with a high risk for hypomagnesaemia, such as those using diuretics, or those suffering from alcoholism or persistent diarrhea were excluded, as well those with a confirmed diagnosis of diabetes.

The primary trial end point was the change in HOMA-IR index. Sample size was determinate by a statistical power of 80%, alpha value 0.05, and allowing non-improve in the HOMA-IR of 40 and 80% for the patients receiving Mg and placebo supplementation. The required sample size to detect a treatment effect was 26 patients in each group. Because of possible lost of follow-up, 35 patients were enrolled in each group.

### Clinical and laboratorial evaluation

**Figure 1:**
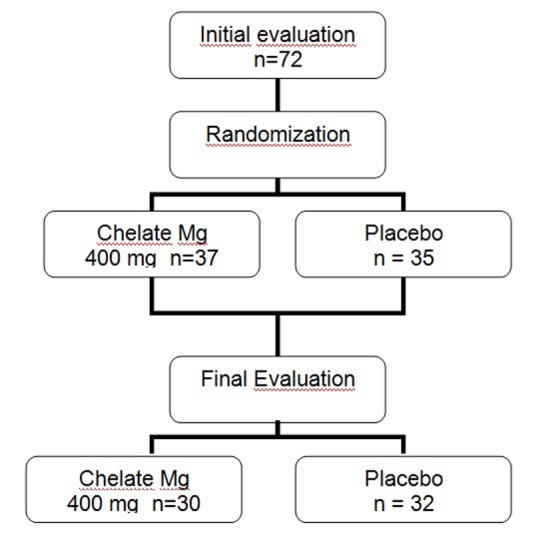
Study design.

The patients were initially subjected to a clinical and laboratory evaluation and then randomized to receive placebo or 400 mg/day of chelated Mg (divided in two daily doses for 12 weeks). All subjects received patronized nutritional recommendation and an exercise program for sessions at least three times a week, and they were instructed to not change the diet nor exercise program. Clinical evaluation was performed after 45 and 90 days after the baseline period. Laboratory assessment was done 90 days after Mg replacement. The study design is shown in Figure 1.

Compliance treatment was assessed by counting pills. All subjects gave their informed consent, and a local institutional ethics committee approved the study protocol.

The clinical evaluation included body mass index (BMI), calculated as body weight (kg) divided by height (m) squared; waist circumference, measured at the middle point between the iliac crest and the last costal arch, at minimal expiration, and in a supine position; blood pressure, measured twice. Achantosis nigricans and signs of inflammation were described if present.

All measurements and blood collection were performed in the morning following a 12-h fast. The laboratory tests performed were fasting blood glucose, cholesterol, triglycerides, HDL cholesterol, uric acid, AST, ALT, γGT by dry chemistry (VITRUS 950 - 250), insulin by chemiluminescence’s spectroscopy, high sensitivity C-reactive protein (CRP) by nephelometry, magnesium in serum (SMg) and in mononuclear cells (MMg) by atomic absorption spectrophotometry (VARIAN - 220).

The homeostasis model assessment (HOMA-IR) was used as a measure of insulin resistance (HOMA-IR = insulin (mU/L) × glucose (mmol/L)/22.5) [12]. Insulin resistance was considered when HOMA-IR was higher than 2.7 [13]. Mononuclear cells separation was previously described [8]. The final suspension consisted of a mean of 97.5% lymphocytes, 2.3% monocytes and 0.15% neutrophils. MMg was expressed by cell proteins, which were measured using the Follin phenol reagent as described by Rodrigues et al [14].

### Statistical analysis

Continuous quantitative data are expressed as means ± standard deviation (SD). Two-tailed parametric tests were used for comparison of normally distributed variables, and non-parametric tests to compare variables when the assumption of normal distribution was not met. Student’s paired *t*-tests or Wilcoxon tests were performed before and after treatment for comparison, and for non-paired comparison, independent *t*-tests or Mann-Whitney tests were used. Categorical variables were compared by Chi-squared or Fischer exact tests. A two-tailed P value ≤ 0.05 was considered statistically significant. Analysis was conducted with the intention to treat.

Data analysis was performed using the “Statistical Package for the Social Science” (SPSS, Inc., Chicago, IL, USA) 16.0 version.

## Results

Seventy-two women with MS without diabetes were enrolled, aged 45.7 ± 11.8 years. Baseline characteristics were not statistically different between the groups (Table 1).

**Table 1:** Baseline Characteristics of Mg and Placebo Groups

	Placebo (n = 37)	Magnesium (n = 35)
Age (years)	46.6 ± 12.3	44.6 ± 9.7
BMI (kg/m^2^)	35.1 ± 6.3	35.5 ± 8.2
Waist (cm)	107.5 ± 12.5	< 80
Systolic blood pressure (mm Hg)	134 ± 17	134 ± 15
Diastolic blood pressure (mm Hg)	86 ± 10	85 ± 8
Glycemia (mg/dL)	99 ± 10	103 ± 15
HOMA-IR	3.31 ± 1.86	3.41 ± 2.03
Insulin (mU/L)	13.4 ± 6.4	14.5 ± 7.6
Cholesterol (mg/dL)	190 ± 46	203 ± 46
Triglycerides (mg/dL)	137 ± 61	136 ± 54
HDL-c (mg/dL)	43 ± 10	46 ± 10
LDL-c (mg/dL)	120 ± 39	129 ± 43
GGT (mg/dL)	44 ± 51	32 ± 19
PCR (mg/L)	6.7 ± 7.0	5.8 ± 5.3
SMg (mg/dL)	1.80 ± 0.22	1.79 ± 0.15
MMg (µg/mg)	0.89 ± 0.43	0.90 ± 0.40

At baseline, there were no differences for any parameters between groups.

Hypomagnesemia was identified in 23.2% of patients and intracellular depletion in 36.1%. Insulin resistance (HOMA-IR ≥ 2.7) was observed in 36 patients (50%). The MMg means were lower in patients with obesity (0.94 ± 0.54 μg/mg vs. 1.19 ± 0.6 μg/mg, P = 0.04), and insulin resistance (0.84 ± 0.33 μg/mg vs. 1.14 ± 0.69 µg/mg, P < 0.05).

Sixty-one patients completed treatment, and nine did not return to visits, or stopped treatment without an apparent reason. One patient was excluded in Mg group because of urticary issues.

The mean of weight loss in whole patients was 1.9 ± 1.7 kg (median, 1.7 kg), with no significant difference between Mg and placebo group. They reduced waist circumference and BMI in the same proportions. Table 2 shows Mg concentrations and metabolic parameters before and after treatment.

**Table 2:** Pre- and Post-Treatment Evaluation

	Magnesium group (n = 30)			Placebo group (n = 32)		
	Before treatment	After treatment	P value	Before treatment	After treatment	P value
Weight (kg)	84.6 ± 17.2	82.6 ± 17.7	0.001	89.1 ± 18.6	86.2 ± 19.6	0.018
BMI (kg/m^2^)	33.7 ± 6.7	32.8 ± 6.9	0.001	34.9 ± 6.1	34.2 ± 6.1	0.020
Waist (cm)	104 ± 12	101 ± 10	0.001	108 ± 12	106 ± 12	0.001
Systolic blood pressure (mm Hg)	134 ± 12	124 ± 27	0.004	132 ± 17	128 ± 19	0.293
Diastolic blood pressure (mm Hg)	86 ± 7.6	82.9 ± 8.9	0.452	85 ± 10	83 ± 13	0.454
Glycemia	104 ± 16	102 ± 12	0.091	99 ± 11	100 ± 10	0.545
Insulin (mU/L)	12.3 ± 7.0	10.7 ± 6.3	0.240	14.4 ± 6.2	12.6 ± 6.5	0.134
HOMA-IR	3.2 ± 2.0	2.8 ± 1.9	0.368	3.6 ± 1.9	3.2 ± 1.8	0.337
Cholesterol (mg/dL)	204 ± 46	208 ± 38	0.471	192 ± 47	191 ± 42	0.940
HDL-c (mg/dL)	46.5 ± 9.0	46.3 ± 10.8	0.857	41.9 ± 10.2	41.4 ± 8.5	0.722
LDL-c (mg/dL)	127 ± 42	139 ± 38	0.238	120 ± 41	127 ± 41	0.173
Triglycerides (mg/dL)	127 ± 43	131 ± 41	0.579	144 ± 64	136 ± 73	0.431
Uric acid (mg/dL)	4.9 ± 1.0	4.9 ± 1.1	0.945	5.6 ± 1.5	5.5 ± 1.8	0.448
PCR (mg/L)	6.0 ± 5.3	6.1 ± 6.5	0.991	6.3 ± 7.9	5.3 ± 3.3	0.463
SMg (mg/dL)	1.82 ± 0.14	1.81 ± 0.16	0.877	1.87 ± 0.18	1.75 ± 0.21	0.073
MMg (µg/mg)	0.90 ± 0.40	1.21 ± 0.73	0.089	0.88 ± 0.37	0.86 ± 0.23	0.920

Serum and intracellular Mg did not change in intervention nor in placebo group (1.82 ± 0.14 mg/dL to 1.81 ± 0.16 mg/dL, P = 0.89 and 0.90 ± 0.40 μg/mg to 1.21 ± 0.73 μg/mg, P = 0.089). Systolic blood pressure reduced significantly only in the Mg group (134 ± 12 mm Hg vs. 124 ± 27 mm Hg, P = 0.004 and 132 ± 17 mm Hg vs. 128 ± 19 mm Hg, P = 0.293), and occurred in a tendency to reduction in glucose levels (104 ± 16 mg/dL to 102 ± 12 mg/dL, P = 0.091 vs. 99 ± 11 mg/dL and 100 ± 10 mg/dL, P = 0.545). In spite of the blood pressure fall in Mg group, the compared means changes after treatment between the two groups did not shown significant difference (Table 3).

**Table 3:** Means Changes After Treatment

	Magnesium	Placebo	P
Δ SBP (mm Hg)	-8.5 ± 27.8	-4.0 ± 19.7	0.498
Δ DBP (mm Hg)	-2.0 ± 9.3	-1.9 ± 13	0.964
Δ Fast blood glucose (mg/dL)	-2.0 ± 6.2	+0.9 ± 8.3	0.129
Δ Insulin	-1.7 ± 6.4	-1.6 ± 7.2	0.938
Δ HOMA-IR	-0.36 ± 2.10	-0.32 ± 1.80	0.928
Δ Triglycerides (mg/dL)	+3.6 ± 35	-8.1 ± 55.3	0.337
Δ HDL (mg/dL)	-0.17 ± 5.1	-0.50 ± 7.6	0.847

Δ: means change from baseline to end point between the beginning and the end of study; SBP: systolic blood pressure; DBP: diastolic blood pressure.

HOMA-IR did not reach statistically significant reduction in the both groups (3.2 ± 2.0 to 2.8 ± 1.9, P = 0.368 in intervention group, and 3.6 ± 1.9 to 3.2 ± 1.8 in placebo group). The other variables did not alter with treatment.

The Mg supplement was well tolerated, and there were no relevant side effects, which occur in same proportion in patients taking Mg or placebo: slight epigastric pain (5.4% vs. 8.6%), nausea (2.7% vs. 5.7%), and diarrhea (2.7% vs. 2.9%), which did not require treatment or interruption of medications.

## Discussion

The present study showed a prevalence of hypomagnesemia in 23.2% of patients with MS without diabetes, and intramononuclear depletion in 36.1%. This frequency of hypomagnesemia was higher when compared with healthy people (3.6%) [8] and with hospitalized patients (10%) [15], but lower than that found in by Guerrero Romero in patients with insulin resistance (65.6%) [7].

There is no reported data about intracellular Mg depletion in patients with MS. In this study, the frequency of intramononuclear depletion was high, and more pronunciated in patients with obesity. Elsewhere, an independent association with BMI was shown. The low levels of intracellular Mg observed in patients with insulin resistance, present in 50% of these patients, suggest a potential mechanism to explain this elevated prevalence of intracellular Mg depletion. Insulin has specific ionic effects to stimulate the transport of Mg from the extracellular to the intracellular compartment, with unclear mechanism [16].

Other postulated mechanism to explain the high prevalence of Mg depletion, could be the low ingestion of Mg rich foods by obese people, as described by others [6, 17]. Unfortunately we did not measure the Mg intake accurately to test this hypothesis.

Mg replacement was well tolerated, with few side effects, comparable to the placebo group. However, after 3 months 400 mg/day Mg supplementation, the serum and intracellular levels after did not increase significantly as was expected, a fact that may be explained by the insulin resistance present in the majority of the patients studied, impairing Mg entrance in the cells. The recommended dietary allowance for Mg for men and for women is 420 and 320 mg/day respectively [18]. The tolerable upper daily intake for supplement in adults is 350 mg according to NIH, but the supplement dose in many studies varies from 200 to 630 mg/day [10, 19]. Probably, in these patients with insulin resistance, higher doses of supplements may be necessary to correct intracellular Mg depletion.

Oral Mg supplements combine Mg with another substance as a salt (chloride, carbonate), oxide, or it can be chelated by amino acids. The amount of Mg in a compound and its bioavailability influences the effectiveness of the Mg supplement. Mg chloride or Mg lactate for example, has higher bioavailability than Mg oxide [20]. The best form for Mg replacements seems to be chelated Mg, since it is not affected by the acidic pH of the stomach; fats and fibers do not interfere with its absorption, and the transport across the mucosa cell do not require vitamin intervention, as occurs with same metal ions absorption [21]. For these reasons, it was the form of replacement chosen in this study.

The importance of Mg on insulin sensitivity has been studied by many authors in diabetic patients [22, 23], and in non-diabetic patients [24]. Mg is essential for the phosphorilation of tyrosine kinase on the insulin receptor, as well as for other protein kinases involved in post-receptor insulin signaling [16]. For these reasons, Mg replacement may contribute to reducing insulin levels and fasting glucose levels.

In this study, however, after 12 weeks of taking chelated Mg (400 mg/day) or placebo, while following a hypo-caloric diet and an exercise program, patients lost weight and reduced their waist circumference in both groups, but HOMA-IR did not show significant variation during the period of study. A fall in systolic blood pressure and a tendency to reduce glucose levels was seen only in the Mg group, but the mean changes comparison between groups at the end of treatment was not significantly different. On the other hand, Hadjistavri et al, using a high dosage of Mg pidolate (600 mg), in hypertensive patient, showed a benefic effect in insulin resistance after 3 months supplementation [25].

The linkage between Mg deficiency and hypertension was first demonstrated in the Honolulu Heart Study [26], and was confirmed by prospective studies, including the cross-sectional study Atherosclerosis Risk in Communities (ARIC) study, which demonstrated a negative correlation of dietary and serum Mg levels with systolic and diastolic pressure [27]. Mg influences blood pressure regulation by modulating vascular tone and reactivity [28].

A limitation of this study deserves to be mentioned. We did not select patients with insulin resistance nor with Mg deficiency, a fact that can explain the difference in the results when compared with data of Guerrero-Romero who showed an improvement in insulin resistance in Mg deficient patients with insulin resistance, using Mg chloride, 2.5 g/day, what corresponds to 630 mg of elementary Mg, a higher dosage than the one used in this study, other difference that can explain the divergent results.

In conclusion, we demonstrate that a decrease in serum and in intracellular Mg concentration is common in patients with MS, especially in those with obesity and insulin resistance, but Mg replacement with 400 mg/day of chelated Mg for 3 months did not correct Mg depletion neither improving insulin resistance nor metabolic control.

As insulin resistance can make Mg entrance into the cells difficult, more prolonged replacement in doses higher than usual are probably needed in order to establish if routine Mg replacement or selective administration in patients with MS, could improve metabolic control or prevent diabetes and cardiovascular diseases.
